# Development of organo-bioformulations for improved phosphorus availability and wheat productivity: insights from adsorption isotherms and field validation

**DOI:** 10.3389/fmicb.2026.1848421

**Published:** 2026-07-09

**Authors:** Rafia Mahmood, Saira Tabbasum, Muhammad Akhtar, Nimra Tahreem, Khansa Ejaz, Naima Mahreen, Waqar Younis, Muhammad Imtiaz, Umer Zeeshan Ijaz, Sumera Yasmin

**Affiliations:** 1 Soil and Environmental Biotechnology Division, National Institute for Biotechnology and Genetic Engineering College, Pakistan Institute of Engineering and Applied Sciences (NIBGE-C, PIEAS), Faisalabad, Pakistan; 2 Soil and Environmental Sciences Division, Nuclear Institute for Agriculture & Biology - College, Pakistan Institute of Engineering & Applied Sciences (NIAB-C, PIEAS), Faisalabad, Pakistan; 3 University of Veterinary and Animal Sciences Lahore, Sub-campus Jhang, Lahore, Pakistan; 4 James Watt School of Engineering, University of Glasgow, Glasgow, United Kingdom

**Keywords:** organo-bioformulation, phosphorus adsorption dynamics, plant-microbe interactions, soil health, soil nutrient management, sustainable wheat productivity, yield stability

## Abstract

**Introduction:**

Low phosphorus (P) availability is a major constraint to crop productivity in calcareous soils due to higher P sorption and fixation processes. In these soils, applied phosphorus (P) is rapidly immobilized through precipitation with calcium and adsorption onto CaCO₃ surfaces, resulting in low P availability. Therefore, to mitigate this issue, the current research is focused on the development of organo-bioformulation and quantifying P sorption from diammonium phosphate (DAP) and organo-bioformulations to estimate the fertilizer requirement to maintain an optimal soil solution P concentration.

**Methods:**

Phosphate-solubilizing bacteria (PSB) were isolated from wheat rhizosphere soil collected from drought-prone regions of South Punjab. The isolates were characterized for plant growth-promoting traits and demonstrated high efficiency in solubilizing tricalcium phosphate (TCP) (up to 448 μg/mL), along with production of plant growth regulators such as indole-3-acetic acid (IAA) (up to 402 μg/mL), organic acids, siderophores, ACC deaminase activity and also exhibited zinc-solubilizing potential. Well-characterized PSB strains were formulated into consortia and used to develop two organo-bioformulations using Filter Mud (FM) and Cow Dung (CD) with 5% sodium alginate as a natural adhesive. Microcosm studies using Freundlich isotherms showed reduced P adsorption and enhanced P availability compared to diammonium phosphate (DAP).

**Results:**

To maintain a critical soil solution P level (0.2 mg L^−1^), DAP required 166.96 kg P₂O₅ ha^−1^, whereas requirements decreased to 129 kg ha^−1^ and 111 kg ha^−1^ with filter mud and cow dung-based organo-bioformulations, respectively. *In planta* evaluation on wheat crop showed that both organo-bioformulations significantly improved plant height up to 26%, biomass (24%), grain yield (16%), and grain P (53%) compared to the 80% uninoculated P fertilizer control. Microplot and field trials confirmed these findings, promoting vegetative growth, plant nutrient uptake, and elevated soil phosphatase activity. In field conditions, plant grain yield improved up to 17% and grain P up to 21% over 80% P control. Principal component analysis further confirmed positive effects on nutrient uptake, vegetative growth, and yield.

**Conclusion:**

Thus, these bioformulations provide a sustainable solution for nutrient management in calcareous soils, reducing chemical fertilizer use and supporting long-term soil health and crop productivity.

## 1 Introduction

Intensive modern agricultural systems, particularly those influenced by the Green Revolution, prioritize higher production while often neglecting long-term soil health and fertility (Jamal et al., 2023). This problem is particularly pronounced in calcareous soils, where alkaline pH and high calcium carbonate (CaCO₃) content interact synergistically with low soil organic matter (2.6 to 6.4 mg C g^−1^) levels to exacerbate nutrient immobilization, especially phosphorus (P) (Leytem and Mikkelsen, 2005). Calcareous soils, which span approximately 800 million hectares, primarily in arid and semi-arid regions, form precipitation with Ca^2+^ and Mg^2+^ ions and by sorption on calcite surfaces (16 to 200 m^2^ g^−1^) (Adnan et al., 2022), forming stable, insoluble calcium phosphate minerals such as dicalcium phosphate (CaHPO₄) and hydroxyapatite (Ca₅(PO₄)₃OH) (Brownrigg et al., 2022), leaving less than 10–20% of applied P in bioavailable form that crops can uptake (Adnan et al., 2022). Wheat (*Triticum aestivum L.*), a major staple crop globally, is grown on approximately 9.1 million hectares in Pakistan, producing approximately 25 million tons annually, while global wheat production exceeds 770 million tons per year (Ray et al., 2022). Thus, the continuous application of soluble P fertilizers, while aimed at boosting short-term productivity, has instigated a vicious cycle of soil degradation and resource waste (Spohn et al., 2023). This practice depletes soil organic matter (SOM), a cornerstone of soil fertility that acts as a natural buffer against P fixation by competing for sorption sites (Vermeiren et al., 2021). For instance, a meta-analysis by McDonald et al. (2023) reported that in calcareous soils of South Asia, wheat yields were 15–25% lower than their potential primarily due to P deficiency, despite routine P fertilizer application (Gao et al., 2023). Due to higher P sorption in calcareous soils severely restricting availability to plants, reducing crop productivity, and driving excessive reliance on chemical fertilizers, leading to negative environmental impacts of chemical fertilizers, including eutrophication, soil carbon footprint, and resource depletion (Memon et al., 2024). Therefore, the development of sustainable nutrient management strategies enhances nutrient bioavailability and promotes long-term agroecosystem sustainability.

A cornerstone of this sustainable ecological approach is the mobilization of the soil’s innate microbial workforce. The soil microbiome, particularly phosphorus-solubilizing bacteria (PSB), offers a biological key to unlock the fixed P reserves. PSB genera such as *Pseudomonas*, *Bacillus*, and *Rhizobium* employ several mechanisms to solubilize insoluble P (Ouf et al., 2023; Khan et al., 2025), including the secretion of organic acids with low molecular weight, e.g., gluconic, citric, and oxalic acids, that acidify the micro-environment and chelate Ca^2+^ ions (Kiprotich et al., 2025), production of inorganic acids (e.g., H₂SO₄ and HNO₃), which lower soil pH that enhance the solubility of mineral phosphates, and the enzyme-mediated mineralization of organic P via phosphatases (Hussain et al., 2025; Sharma et al., 2013). Various studies showed that inoculation of PSB (*Pseudomonas and Bacillus*-based) in a calcareous soil significantly increased available P and crop yield by enhancing rhizosphere acidification and phosphatase activity as compared to uninoculated controls (Parvage et al., 2012; Khan A. et al., 2023). When incorporated into organic fertilizers, PSB act as “miners’ agents” that enhance P bioavailability (Othman and Panhwar, 2014). The efficacy of PSB is significantly enhanced when delivered in an organic carrier, such as filter mud and compost. This creates a synergistic bioformulation where organic carriers provide a rich source of carbon and energy, fostering a thriving microbial community (Khan A. et al., 2023; Khan K. S. et al., 2023). It improves soil structure and provides organic acids that can directly chelate cations and facilitate P release. Previously documented that the combined application of PSB and compost not only increased soil available P up to 50% but also elevated SOM content by 1.2% over two cropping seasons, demonstrating a dual benefit for nutrient availability and soil carbon sequestration (Gao et al., 2023; Khan et al., 2022).

Despite the great potential to improve the efficiency of nutrient use and soil health, the widespread adoption of biostimulants/biofertilizers still faces major challenges that restrict their use in agriculture. Various microbial formulations have been designed and developed by using liquid or solid carrier materials. However, liquid formulation often exhibited a rapid decline in microbial viability and its metabolic activity following the application of cell suspensions into the soil, particularly in the absence of appropriate additives (Bashan et al., 2016; Bashan et al., 2014). These challenges are often exacerbated under field conditions where desiccation, temperature fluctuations, and osmotic stress severely restrict microbial survival and rhizosphere establishment (Mitter et al., 2021; Malusá et al., 2012). Due to this reason, different commercial bioformulations often have an inconsistent performance under field conditions relative to outcomes obtained under laboratory or controlled experiment conditions (Stamenković Stojanović et al., 2018; Vassilev et al., 2020).

To address these limitations, increasing emphasis has been given on solid formulations utilizing organic and inorganic carrier material, developed in solid, or powdery forms, and systematically classified according to their particle sizes or method of application (Malusá and Vassilev, 2014; Stamenković Stojanović et al., 2018). Solid formulations provide physical protection to microbial cells and create a buffered microenvironment that enhances shelf life, stress tolerance, and gradual release of nutrients in soil. Several studies have reported that solid organic formulations maintain higher viable cell counts over extended storage periods and improve root colonization and functional performance of phosphate-solubilizing bacteria (PSB) compared with liquid inoculants (Bansal et al., 2025; Shravani et al., 2019). The most importantly used carriers in solid formulations are agro-industrial wastes, compost, peat, perlite, vermiculite, rock phosphate, calcium sulfate, and polysaccharides (Sahu and Brahmaprakash, 2016; Qadir et al., 2024). More recently, agro-industrial by-products have gained attention as alternative organic carriers due to their low cost, high organic carbon content, and compatibility with microbial inoculants (Vassilev et al., 2020; Bashir et al., 2024). These materials contribute to soil organic matter, microbial activity, and nutrient mobilization, thereby supporting sustainable agricultural systems (Bashir et al., 2024). Hence, the primary objective of this study was to develop and optimize a successful solid organo-bioformulation based on PSB consortium and agro-industrial by-products as an exclusive carbon source and organic amendments, while ensuring optimal shelf life. We anticipated that this strategy would be efficient in producing a sustainable and effective alternative to traditional mineral P fertilization.

Despite the agronomic benefits of PSB and organic amendments, a critical mechanistic gap persists in our understanding of how a unified PSB-based organo-bioformulation alters the fundamental P sorption–desorption processes in calcareous soils. Most studies focus on short-term plant uptake or extractable P changes but provide limited insight into the mechanistic P retention and release behavior (Nair and Reddy, 2013). As a result, questions persist regarding how long organic–inorganic amendments can sustain the elevated P availability, or how they influence the equilibrium between labile and fixed P in calcareous soils (Oldfield et al., 2019; Gichangi, 2019). This lack of mechanistic understanding has been highlighted in recent research; for example, Raymond et al. (2023) reported that even in well-studied soils, the availability of fertilizer P after application is often “poorly understood,” owing to the complexity of soil P reactions (Duminda et al., 2016). Elucidating how PSB cultures and organic amendments mechanistically alter P equilibrium, particularly sorption–desorption kinetics, will be crucial for devising fertilization strategies that reconcile immediate productivity with long-term soil stewardship.

However, different sorption isotherm models such as Langmuir/Freundlich and Temkin offer a tool to untangle this complexity by quantifying soil P binding characteristics, limiting predictive fertilization strategies (Duminda et al., 2016; Singh et al., 2005). The amount of phosphorus (P) required to reach a desired concentration in the soil solution can be more accurately estimated using phosphorus sorption isotherms, as described by the Freundlich adsorption isotherm equation: P = aC^a/b^ (Oldfield et al., 2019; Naeem et al., 2013). In this model, the equilibrium solution concentration (Cp) represents the phosphorus remaining in solution after the adsorption equilibrium is reached (Morgan et al., 2018), while the difference between added P and Cp indicates the amount of P sorbed by soil particles (Bhattacharyya et al., 2015). Thus, the Freundlich model is an empirical model to determine the soil P sorption behavior and fertilizer requirements to maintain sufficient available phosphorus to plants in the soil (Amarh et al., 2021).

To overcome these knowledge gaps, the current research study aimed to explore the potential of phosphate-solubilizing bacteria based organo-bioformulations to regulate phosphorus sorption behavior and enhance phosphorus availability in calcareous soils. PSB isolated from the rhizosphere soils of wheat were characterized for plant growth-promoting (PGP) traits and incorporated for the development of organo-bioformulations by using locally available organic carrier material. The Freundlich sorption isotherm was applied to quantify the soil P absorption and fertilizer requirement to maintain optimal levels of solution P in soil. We hypothesized that PSB-based organo-bioformulations would reduce P fixation, modify P sorption behavior compared with the conventional chemical fertilizers, and enhance P availability, plant nutrient uptake, and wheat productivity. This study, which also gives a mechanistic understanding of P dynamics, describes a sustainable approach to minimize chemical fertilizer inputs and enhance yield in calcareous soils.

## 2 Materials and methods

### 2.1 Collection of soil samples

Soil samples from the rhizosphere were obtained at a depth of 0–20 cm from various crops in drought-stressed areas of South Punjab, Pakistan, i.e., Khanewal, Multan, Lodhran, Bahawalpur, Faisalabad, Sheikhupura, Sahiwal, Bahawalnagar, and Layyah (Table 1). Rhizosphere soil samples were transported to the laboratory for the isolation of PSB and stored at 4 °C.

**Table 1:** Morpho-physiological characterization and plant growth-promoting traits are produced by phosphate-solubilizing bacteria.

Isolated strains	Isolation site	Colony morphology	Colony morphology	Cell morphology	Organism identified	Accession number	Siderophore production^a^	ACC deaminase activity^b^	Zinc solubilization^c^
							+	±	
RM-1	Khanewal	Small, white, dry, umbonate, and convex	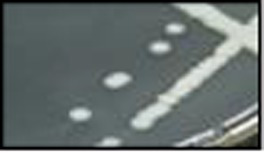	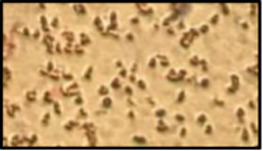	*Acinetobacter baumannii*	PX458093	+	−	8.75
RM-2	Bahawalpur	Large, undulate, convex, creamy, and shiny	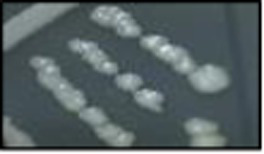	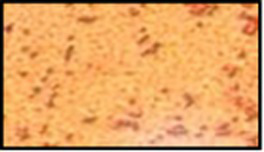	*Enterobacter bugandensis*	PX458094	+++	+	11
RM-3	Bahawalpur	Medium, round, entire, convex, white, and smooth	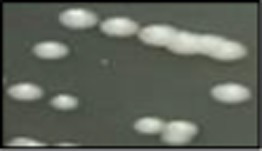	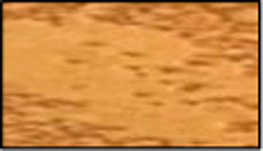	*Acinetobacter pittii*	PX458095	++	+	5.75
RM-4	Bahawalpur	Grayish white, circular, large, smooth, and even transparent	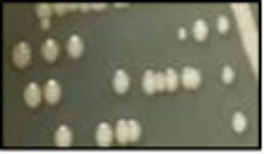	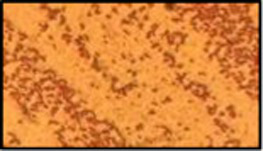	*Klebsiella quasipneumoniae subsp.*	PX458096	+++	+	7.7
RM-5	Multan	Large, irregular, undulate, convex, and creamy	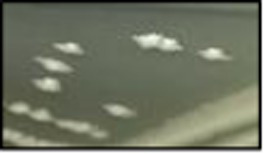	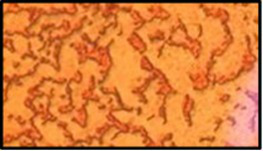	*Enterobacter cloacae*	PX458097	+++	+	3.5

a Siderophore production activity of PSB was quantitatively studied by using the universal CAS assay. + represents siderophore production level.

b ACC deaminase activity was evaluated by isolates’ growth in vials containing 0.5 M ACC.

c Zinc solubilization potential assessed by plate assay, added with ZnO solubilization index (SI).

### 2.2 Isolation of phosphate-solubilizing bacteria

Soil samples were subjected to serial dilutions up to 10^4^, and 100 μL from each dilution was spread onto LB agar plates, followed by incubation at 30 ± 2 °C for 24–28 h. The phosphate solubilization potential of bacterial colonies was analyzed through *in vitro* screening using NBRIP, which uses TCP as an insoluble source of phosphorus. Morphologically, different colonies were streaked on the NBRIP agar plate and incubated at 30 ± 2 °C for 7 days. Plates were observed for the development of halo zones around bacterial colonies. Phosphate solubilization index was calculated by the following equation (Nautiyal, 1999).


$$\begin{array}{l} \text{Phosphate solubilization index}\;(\mathrm{SI})=\\ (\text{diameter of zone clearance})/\text{diameter of growth}. \end{array}$$

### 2.3 Quantitative of phosphate-solubilizing activity and organic acid production

Phosphate-solubilizing ability of bacteria was quantified by using the phospho-molybdate blue color method (Suleman et al., 2018). Each freshly grown pure isolated bacterial colony was inoculated separately into NBRIP broth medium. Isolated bacterial culture in NBRIP broth was incubated on a shaker at 150 rpm for 14 days. At 7 days post-inoculation (DPI) and 14 DPI, the culture was centrifuged at 4 °C for 10 min at 4,000 rpm in a sterile Falcon tube to collect a cell-free supernatant. The pH of the supernatant was determined using a pH meter (PHS-3C, REX, Shanghai). To determine soluble phosphorous, 10 mL of distilled H_2_O, 1 mL of supernatant, and 4 mL of reagent B (containing ammonium molybdate, sulfuric acid, ascorbic acid, and antimony potassium tartrate) were added and allowed to react at room temperature for 20 min for color development. A double-beam UV visible spectrophotometer (CamSpec M-350, UK) was used to record the optical density (OD) at 882 nm. The soluble P was determined by plotting a KH_2_PO_4_ standard curve with various standards of 2 ppm to 20 ppm.

Quantification of organic acids in NIBRIP filtrate was performed using high-performance liquid chromatography (HPLC; Agilent 1200 Series; Agilent Technologies, Santa Clara, CA, United States) using a C-18 column. The system was operated with methanol: phosphate buffer (90: 10 v/v; pH 2.7) as mobile phase at a flow rate of 1 mL min^−1^ and monitored at 210 nm (Tahir et al., 2013). Organic acids, i.e., indole acetic acid, acetic acid, malic acid, gluconic acid, gibberellic acid, oxalic acid, citric acid, and succinic acid, were analyzed. Peak area and retention time were compared to standards (Sigma) for the quantification of organic acids (Vyas and Gulati, 2009).

### 2.4 Light microscopy and molecular identifications of isolated PSB

Stereo microscopy and light microscopy were used for cell and colony morphology as well as gram staining of bacterial isolates. The forward primer, fD1, and reverse primer, rD1, with the sequence 5′-AGACTTTGATCCTGCTCA-3′ and 5′-AAGGAGGTGATCCAGCC-3′, respectively, were utilized for amplification of the 16S rRNA gene. These primers have been previously reported to be efficient for amplification of the 16S rRNA gene for various bacterial species (Bahram et al., 2019). The PCR conditions included denaturation at 95 °C for 60 s, annealing at 58 °C for 60 s, and extension at 72 °C for 60 s. A final extension step was performed at 72 °C for 10 min following the completion of the amplification cycles. The amplified products of PCR were loaded onto 1% agarose gel for visualization of bands. PCR product was purified, and DNA sequencing was carried out commercially from Macrogen Inc., Seoul, South Korea, and the sequences were further analyzed and compared with typed strains with Basic Local Alignment Search Tool (BLAST). Subsequently, sequences were submitted to the National Centre of Biotechnology Information (NCBI), and the allocated accession numbers in GenBank^1^ are provided in Table 1.

### 2.5 Bioassays for detecting growth-promoting activities of bacterial isolates

Zinc (Zn) solubilization potential of isolated strains was evaluated using a Tris-minimal agar medium containing insoluble zinc oxide (ZnO). Halo-zone formation surrounding PSB colonies indicated Zn solubilization activity of bacterial isolates. Chrome Azurol S (CAS) agar medium was used to identify siderophore-producing PSB, indicated by the development of a pink color around the bacterial colony of PSB (Schwyn and Neilands, 1987). The nitrogen-fixing potential of the bacterial isolates was assessed based on their growth on a semi-solid N-free medium (Hardy et al., 1968) in glass vials and incubated at 28 °C for 7 days. IAA production was evaluated by using the colorimetric technique (Kuźniar et al., 2021). Bacterial cultures were incubated in LB broth containing 0.01% tryptophan for 14 days at 110 rpm on an orbital shaker. IAA production was detected by using Salkowski’s reagent (0.5 M FeCl_3_ + 35% H_2_SO_4_). An aliquot of 100 μL culture was treated with Salkowski’s reagent and incubated at ambient temperature for 30 min. The development of pink color indicated IAA production (Gordon and Weber, 1951).

### 2.6 Development of PSB consortia

For consortia development, four efficient PSB (RM-1, RM-2, RM-3, and RM-4) were studied by agar well diffusion assay (Balouiri et al., 2016). In this assay, LB agar plates were prepared, and a bacterial suspension of one isolate was uniformly spread on the agar surface. Wells (6 mm diameter) were made aseptically. Then, 100 μL of bacterial culture from each tested isolate was added into the wells, and plates were incubated at 28 ± 2 °C for 72 h. The inhibition zone around bacterial colonies was studied for the presence or absence of an inhibition zone.

### 2.7 Optimization and development process of organo-bioformulation

For the selection and optimization of adhesives, different bioformulations were developed by two type of adhesives, i.e., gum Arabic and sodium alginate at different concentration rates, and were screened for their crushing strength and analyzed the size of both bioformulation using ImageJ software. Well diffusion assays were performed to analyze the compatibility of the adhesives with PSB consortia. An incubation study was designed to investigate the survival efficiency of the inoculated PSB in formulations containing different concentrations of sterilized carrier substrates (including cow dung and filter mud) and adhesives (including sodium alginate and gum Arabic). Four treatments were as follows: T1: cow dung + alginate + PSB consortium, T2: Filter mud + alginate + PSB consortium, T3: Cow dung + gum Arabic + PSB consortium, T4: Filter mud + gum Arabic + PSB consortium. Bacterial viability was monitored at regular intervals of 15, 30, and 60 days after inoculation using the spread plate method (Vyas and Gulati, 2009), and viable count (CFU g^−1^) was calculated and plotted on a logarithmic scale. The P solubilization activity of isolates was assessed by spot inoculating colonies on NBRIP agar and incubating at 28 ± 2 °C for 5–7 days to observe halo zone formation.

Two organo-bioformulations were developed using different carrier materials, selected adhesives, and a consortium of isolated PSB (RM-1, RM-2, RM-3, and RM-4) with cell density of approximately 10^8^ CFU/mL for each strain prior to mixing. Filter mud and cow dung as carrier substrate were sieved through a 2 mm sieve and autoclaved. In Bioformulation 1, carrier material: 10 kg autoclaved filter mud, bacterial consortium: 200 mL/kg, and sodium alginate: 5% were added and thoroughly mixed to achieve a uniform consistency. Similarly, in Bioformulation 2 (BF_RC), carrier material: 10 kg cow dung, bacterial consortium: 200 mL/kg, and sodium alginate: 5% were added and mixed thoroughly. The developed organo-bioformulations (filter mud-based bioformulation: FM-BF, cow dung-based bioformulation: CD-BF) were then air-dried, and their crushing strength was assessed using a simple finger test: soft; crushed between forefinger and thumb, medium hard; crushed with finger on hard surface, hard; remained intact after exerting pressure by forefinger on a hard surface (Narayanasamy et al., 2023).

#### 2.7.1 Quality control analysis of organo-bioformulation

The pH and electric conductivity (EC) of both bioformulations were determined using a pH meter (PHS-3C, REX) according to Thomos (Yahya et al., 2021) and EC meter (DDS-307A, REX Shanghai), respectively (Yahya et al., 2021). The organic matter was measured by the wet oxidation method (Shaw, 2006). Total nitrogen and phosphorus were determined by Kjeldahl (Sáez-Plaza et al., 2013) and vanadate-molybdate method (Tandon, 2005), respectively.

#### 2.7.2 Shelf-life assessment

The prepared organo-bioformulations were stored in sterilized zip bags kept at room temperature, and the viability of inoculated PSB was assessed at regular intervals of 15, 30, 60, and 90 days post-inoculation. At each interval, bacterial viable count (CFU g^−1^) was determined from 1 g of bioformulation by using the previously described serial dilution and plating method. The mean values were on a logarithmic scale. Additionally, the recovered strains were further subjected to PGP trait characterization as mentioned in Section 2.5.

### 2.8 Microcosm soil study

This study assesses soil phosphorus adsorption using the Freundlich equation and evaluates how organo-bioformulations reduce P fixation compared to chemical fertilizer and predict fertilizer requirement (DAP).

For experimental analysis, surface soil was collected from the NIBGE field, Faisalabad (31.398035888, 73.02560042). Collected soil was air-dried, sieved through a 2 mm sieve, homogenized, and stored in closed glass jar to minimize moisture loss. The available soil P of soil samples was analyzed by the Olsen method. The study comprised five treatments in triplicate. The experiment was conducted in a glass jar at room temperature during the months of November and December of 2025. To evaluate the effect of bioformulations on soil phosphorus (P) adsorption, five treatments were designed. In the control treatment, 100 ppm P was applied in the form of diammonium phosphate (DAP) solution. In treatments T2 and T3, filter mud-based bioformulation (FM-BF) was applied at rates of 25 and 50 kg/acre, respectively, with the DAP dose adjusted so that the total phosphorus input (DAP + bioformulation) was equivalent to 100 ppm, matching the control. Similarly, treatments T4 and T5 received cow dung-based bioformulation (CD-BF) at 25 and 50 kg/acre, respectively, with DAP adjusted to maintain total P equivalence to the control. The control treatment received only 100 ppm DAP solutions. The soil samples were wetted with distilled water to attain field capacity and equilibrated for 35 days at room temperature. Soil available P was extracted using the Olsen method at 10 DPI and 20 DPI of each treatment. At 35 days post-inoculation (DPI), a series of phosphate solutions (0, 10, 20, 40, and 80 ppm) was prepared from each treatment to construct an adsorption isotherm. From each treatment, 2.5 g of soil sample was added in a series of P solutions and agitated on a mechanical shaker for 24 h. Following equilibration, the suspension was filtered through Whatman number 42 filter paper. Phosphorus concentration in the filtrates was determined using molybdenum blue colorimetric method (Murphy and Riley method) (Hussain et al., 2025). The amount of P adsorbed was calculated by the difference in P concentration of the solutions before and after equilibrium. The sorption isotherms were assessed using the modified Freundlich equation proposed by Le Mare (Sharma et al., 2013) as follows:


$$P=aC^{b/a}$$ (1)

In this equation, *P* represents the amount of phosphorus adsorbed per unit mass of soil (𝜇g mL^−1^), C represents the phosphorus concentration in soil solution, and *a* and *b* represent the amount of *P* adsorbed and buffer capacities. The parameters *a* and *b* were derived using regression analysis to logarithmic transformation of the adsorption isotherms data. Theoretical fertilizer doses for DAP, FM-BF, and CD-BF were computed to achieve the desired soil solution P level of 0.20 mg L−1. Furthermore, regression relationships were developed between calculated quantities of P fertilizer and both organo-bioformulation levels to estimate P fertilizer requirement.

### 2.9 *In planta* evaluation of organo-bioformulation for growth promotion of wheat

#### 2.9.1 Pot experiment

A pot experiment was carried out at NIBGE under net house conditions, during wheat growing season (November–April 2024–2025). To evaluate the impact of both developed organo-bioformulations on wheat variety Arooj using native soil collected from Faisalabad (31.398035888, 73.02560042). Prior to the experiment, seeds were rinsed twice with autoclave-distilled water to attain soil field capacity and each pot (30 cm diameter) was sown with five seeds. This experiment was designed in the form of a completely randomized design (CRD), with six treatments and three replications. Treatments: T1 received 100% of recommended dose of P in form of chemical fertilizer DAP (diammonium phosphate) at 50 kg acre^−1^, T2 received 80% of recommended dosage of P as DAP at 40 kg acre^−1^. Treatments T3 and T4 received filter mud-based bioformulation at 25 and 50 kg acre^−1^ along with the 80% recommended dose of chemical fertilizer, while in T5 and T6, cow dung-based bioformulation was applied at the rate of 25 and 50 kg per acre along with the 80% recommended dose of chemical fertilizer, respectively. Recommended dose of nitrogen (100 kg per acre) in the form of urea was applied to all treatments. Both prepared organo-bioformulations and DAP were added in two split doses. The first dose was applied as a basal dose, and the second was administered with first irrigation. Plants from pots were uprooted 65 days after sowing (DAS) and the harvest stage to evaluate plant growth parameters, plant nutrients (NPK), and chlorophyll content.

The presence of an inoculated PSB was studied using the viable count (Somasegaran and Hoben, 2012) and BOX-PCR by identifying a strain-specific banding pattern (Basheer et al., 2016). Identification of strains was carried out through morphological distinct characteristics and using light microscopy. BOX-A1R primer-based fingerprinting 5′ CTACGGCAAGGCGACGCTGACG 3′ was used to compare re-isolated PSB with their respective pure culture (Basheer et al., 2016).

#### 2.9.2 Field evaluation

To evaluate the effect of developed organo-bioformulations under natural field conditions, a microplot and a field trial were conducted at NIBGE, Faisalabad (31.398008414, 73.02551459), from December 2024 to April 2025. The size of each microplot was 1.5 × 1.5 m^2^, and the field plot was 9 × 9 m^2^. This experiment was designed in the form of CRD, and there are four treatments and replications (4 treatments + 3 replications = 12 entries). Treatments: Treatments: T1 received 100% of recommended dose of P in form of chemical fertilizer DAP (Diammonium phosphate) at 50 kg acre^−1^, T2 received 80% of recommended dosage of P as DAP at 40 kg acre^−1^. In T3, FM-BF and, T4, CD-BF were applied at the rate of 25 kg per acre along with the 80% recommended dose of chemical fertilizer, respectively. Fertilizer application timing was similar as mentioned for the pot experiment.

#### 2.9.3 Physiological parameters

At 65 DAS and the harvest stage, the chlorophyll content of the wheat plant was recorded by using a Soil Plant Analysis Development (SPAD) meter (Süß et al., 2019).

#### 2.9.4 Agronomic and yield parameters

At 65 days after sowing (DAS) and harvest stage, the plants from pots, microplots, and field trial were harvested, and agronomic parameters, i.e., root length, dry weight, shoot, plant biomass, length, fresh weight, number of tillers, and grain yield, were recorded.

#### 2.9.5 Post-harvest nutritional analyses

Plants from pots, microplots, and field trials were uprooted at 65 DAG and harvest stage and analyzed for total plant P content by the vanadate-molybdate method and plant N content by the Kjeldahl method. Exchangeable plant K content was determined by a flame photometer (Asch et al., 2022). Soil alkaline phosphatase activity was determined by the p-nitrophenyl method as described by Tabatabai and Bremner (1969).

### 2.10 Statistical analysis

STATIX software 8.0 (Tallahassee, FL, United States) was used to statistically analyze the experimental data from the field trial, net house experiment, and laboratory scale. For the laboratory scale, net house experiment, and field trial, the least significant difference (LSD) test was applied to compare the means of different treatments at a 5% probability level.

## 3 Results

### 3.1 Isolation of PSB

The rhizosphere soil of different crops was obtained from various regions of South Punjab. The soil samples were investigated for physicochemical properties (Table 1). Out of 181, 17 isolated colonies showed phosphate-solubilizing ability and were further screened for plant growth-promoting attributes. Five isolates, i.e., RM-1, RM-2, RM-3, RM-4, and RM-5, showed significant P solubilization ability, indicated by halo zone formation on NBRIP agar medium. The solubilization index (SI) ranges from 1.8 to 4.7. The highest P solubilization activity was observed by RM-2 (SI: 4.7), followed by RM-3 (SI: 3.5) and RM-4 (SI: 3). Minimum P solubilization activity was observed by RM-5 (SI: 1.6).

#### 3.1.1 Quantitative P solubilization and production of organic acids and plant growth hormone

**Figure 1:**
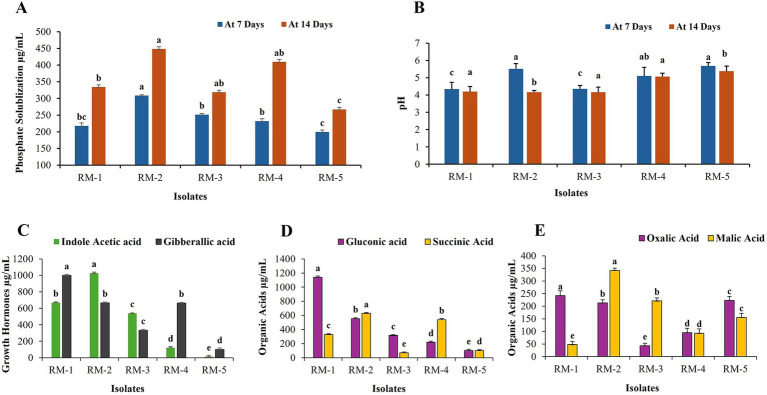
Phosphate solubilization and pH change by PSB. Phosphate solubilization **(A)** and pH change **(B)** at 7 DPI and 14 DPI by rhizosphere bacteria in NBRIP broth added with TCP. **(C)** Production of growth hormone (indole acetic acid and gibberellic acid). **(D)** Organic acid production: gluconic acid, succinic acid, and **(E)** oxalic acid and malic acid by PSB detected by HPLC.

The highest P solubilization activity was shown by the isolates RM-2 (448 μg/mL), RM-3 (418 μg/mL), and RM-4 (409 μg/mL). Minimum P solubilization was observed by RM-2 (234 μg/mL) isolate (Figure 1A). Inoculation with the selected PSB led to a marked reduction in medium pH, ranging from 6.48 to 4.1. A higher decrease (initial pH was 6.47 to 4.1) in pH was observed in the culture filtrate inoculated with RM-1. Minimum drop (initial was 6.47 to 5.38) in pH was observed in RM-5 isolate (Figure 1B).

Organic acids are released in liquid culture based on their P solubilization by PSB strains. High-performance liquid chromatography (HPLC) results highlighted that RM-1 isolate exhibited high levels of growth hormone gibberellic acid (1,000 μg/mL), gluconic acid (1399.3 μg/mL), and acetic acid (1227.3 μg/mL), followed by oxalic acid production (241.8 μg/mL). Higher organic acids produced by RM-2 were gibberellic acid (666.7 μg/mL), malic acid (342.1 μg/mL), oxalic acid (213.1 μg/mL), and acetic acid (69.7 μg/mL). RM-3 produced gibberellic acid (333.3 μg/mL), acetic acid (539.4 μg/mL), malic acid (221.1 μg/mL), and small amounts of gluconic acid (15.5 μg/mL). RM-4 showed minimal organic acid production, gluconic acid (18.6 μg/mL), and acetic acid (121.2 μg/mL). Oxalic acid (222.9 μg/mL) was produced by RM-5. RM-6 exhibited elevated production levels of succinic acid (628.2 μg/mL) and gibberellic acid (663.3 μg/mL) (Figures 1C–E).

#### 3.1.2 Identification of PSB

Selected PSB (RM-1, RM-2, RM-3, RM-4, and RM-5) were grown on an LB plate. Isolate RM-1 formed small colonies, white in color and dry in texture. Bacterial isolates RM-2 and RM-5 formed irregular colonies with convex surfaces and creamy textures. Circular colonies with entire margins were observed for RM-3 and RM-4. All of these strains varied in the color of colonies, with those of RM-3 being white and transparent of RM-4. Gram staining and light microscopy revealed that all isolates (RM-1, RM-2, RM-3, RM-4, and RM-5) were rod-shaped and Gram-negative (Table 1).

Strains were identified as *Acinetobacter baumannii, Enterobacter bugandensis, Acinetobacter pittii, Klebsiella quasipneumoniae subsp.,* and *Enterobacter cloacae.* 16S rRNA gene sequences of *Acinetobacter baumannii as RM-1, Enterobacter bugandensis as RM-2, Acinetobacter pittii RM-3, Klebsiella quasipneumoniae subsp.* RM-4, and *Enterobacter cloacae RM-5* were allocated with NCBI accession numbers of PX458093, PX458094, PX458095, PX458096, and PX458097, respectively. These PSB strains were submitted to NIBGE NBRC, Pakistan (Table 1).

#### 3.1.3 Blood hemolysis test

A blood hemolysis test was performed to screen bacteria for potential biosafety. None of the selected PSB strains lysed or formed halo zones compared to the control, demonstrating that inoculated bacteria may not be pathogenic and may be safe to use to conduct further research (Sáez-Plaza et al., 2013).

### 3.2 Characterization of PSB for plant growth-promoting traits

Zinc solubilization activity was shown by all isolates as indicated by halo-zone formation around. Isolated bacterial colonies on tris minimal salt medium exhibited the solubilization index (SI) ranging from 3.5 to 11 (Table 1). Maximum SI was shown by RM-2 (SI = 11); in contrast, the minimum halo zone formation was observed for RM-5, exhibiting an SI of 3.5.

In the qualitative plate assay, all PSB strains (RM-1, RM-2, RM-3, RM-4, and RM-5) showed siderophore production, as indicated by their growth on CAS agar plate with pink color development around them (Table 1). Among all PSB strains, RM-2 and RM-5 showed relatively high siderophore production. Selected PSB were screened for their plant growth-promoting phytohormone production. RM-1, RM-2, RM-3, RM-4, RM-5, and RM-6 produce IAA (phytohormone) indicated by pink color production by the reaction with Salkowski’s reagent (Table 1). The strains RM-2 exhibited relatively more color development, indicating higher IAA production, followed by RM-4 and RM-5. Contrarily, RM-1, RM-3, and RM-6 produce a pink color with less intensity (Table 1).

Nitrogen fixation activity of selected strains was confirmed by their growth in nitrogen-free media (NFM) and a change in color of medium from green to blue in glass vials inoculated with RM-1, RM-2, RM-3, RM-4, and RM-5. All phosphate-solubilizing isolates exhibited positive ACC deaminase activity, as assessed by their growth in vials supplemented with 0.5 M ACC (Table 1).

### 3.3 Optimization and development process of organo-bioformulation

**Figure 2:**
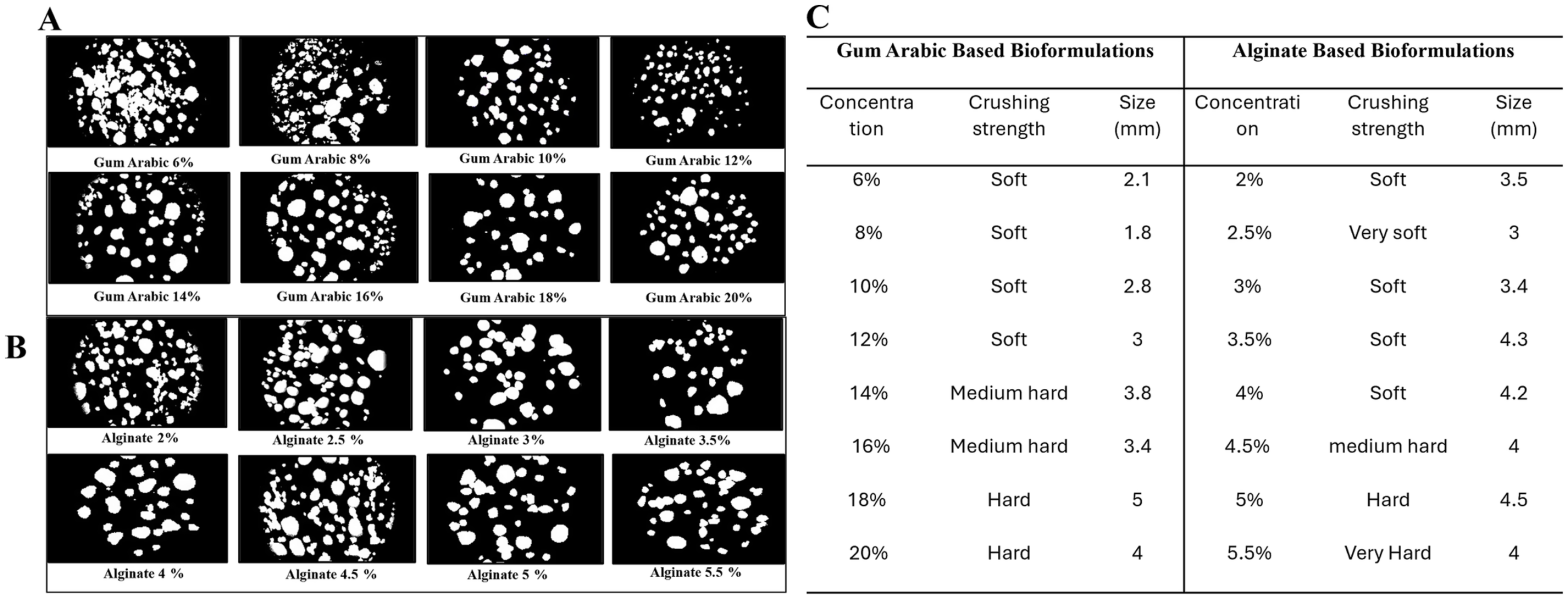
Optimization of adhesives at different concentrations. **(A)** represents different gum Arabic-based formulation, **(B)** represents different alginate-based formulations, and **(C)** shows the composition of bioformulations and their physicochemical characteristics, including concentration of components, size, and crushing strength.

For the development of solid organo-bioformulation, two different adhesives (gum Arabic and alginate) were evaluated at varying concentrations for further final formulation development. Among gum Arabic-based formulations, gum Arabic with 18 and 20% concentrations showed higher crushing strength, followed by 14 and 16% concentrations of gum Arabic having medium crushing strength and a larger size (Figure 2C). Among alginate-based bioformulations, alginate at 2, 3, and 4% concentration exhibited low crushing strength, while alginate at a 5% concentration exhibited the most effective performance (Figure 2C). It produced the highest crushing strength, indicating enhanced mechanical stability, which is considered optimal for handling and field application (Mangwandi et al., 2013) (Figures 2A,B). Thus, two different bioformulations were developed using 5% alginate as the adhesive: one with cow dung as the carrier material, and the other with filter mud as the carrier material, and their effects on wheat growth and soil fertility were evaluated in pot, microplot experiment, and field trials.

#### 3.3.1 Bacterial compatibility study

**Figure 3:**
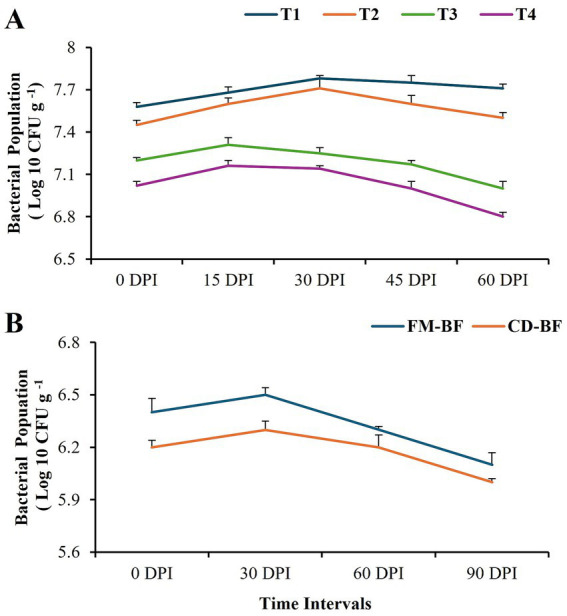
**(A)** Viable count of carrier, adhesive with bacterial compatibility study. Each point reflects the average value of three replicates. Bars show the standard error of means. T1: cow dung + alginate + PSB consortium, T2: Filter mud + alginate + PSB consortium, T3: Cow dung + gum Arabic + PSB consortium, T4: Filter mud + gum Arabic + PSB consortium. **(B)** Viable count of organo-bioformulations at different time intervals. Each point represents the decimal logarithm of viable cells g^−1^ of filter-mud-based bioformulation (FM-BF) and cow-dung-based bioformulation (CD-BF), and it is the mean value of three replicas.

Among the evaluated treatments, T1 (cow dung + alginate + PSB consortium) and T2 (filter mud + alginate + PSB consortium) showed a considerably higher colony-forming unit (CFU g^1^) count during the 60-day post-incubation, highlighting the potential viability and survival of the inoculated PSB (Figure 3A). These findings were further validated through the qualitative phosphate-solubilization assays, which showed that inoculated isolates from each time interval consistently formed halo zones on NBRIP agar, indicating the functional potential of PSB.

In both organo-bioformulations, the viability of inoculated (PSB) was assessed at 15, 30, 60, and 90 days post-inoculation (DPI) by the viable count method. The results showed that the filter mud-based bioformulation consistently had a greater PSB population and survival across all time intervals than the cow dung-based formulation (Figure 3B).

#### 3.3.2 Physicochemical characterization of organo-bioformulation

The physicochemical analysis of both bioformulations revealed that both FM-BF and CD-BF exhibited nearly neutral pH levels. Bioformulation (CD-BF) depicted higher electric conductivity EC (11.5 ms/cm), total dissolved solids TDS (3,000 mg/L), phosphorus (5.7%), and the organic matter (44%), while FM-BF had higher organic content (50%), nitrogen (1.9%), and calcium (0.6%) (Table 2).

**Table 2:** Physicochemical analysis of organo-bioformulations.

Bioformulations	pH	EC (ms/cm)	TDS (mg/l)	Moisture content (%)	P (%)	N (%)	Organic matter (%)	Ca (%)	Mg (%)	S (%)
Filter mud-based bioformulation	6.6	3.0	1,520	3.0	5.4	1.9	50.5	0.6	0.02	0.3
Cow dung-based bioformulation	7.0	11.5	3,000	3.4	5.7	0.9	44.0	0.2	0.05	0.6

### 3.4 Microcosm incubation study

In the microcosm soil study, soil available P was evaluated after 10 DPI and 20 DPI to analyze the efficiency of both organo-bioformulation (FM-BF and CD-BF) in mobilizing fixed P of soil due to applied chemical fertilizer (DAP). After 10 DAI, soil available P was significantly improved in both organo-bioformulation across all treatments. In treatment 4 (35%) supplemented by CD-BF and treatment 3 (28%) increase supplemented by FM-BF was observed, supplemented with CD-BF and FM-BF, respectively, as compared to the control (no inoculation). After 20 DAI, the soil available P content declined across all treatments compared to 10 DAI values. However, overall P availability across all treatments remained high compared to the control. In treatment 4 (28%) and treatment 2 (23%), an increase in available soil P was observed as compared to the uninoculated control treatment (T1).

**Figure 4:**
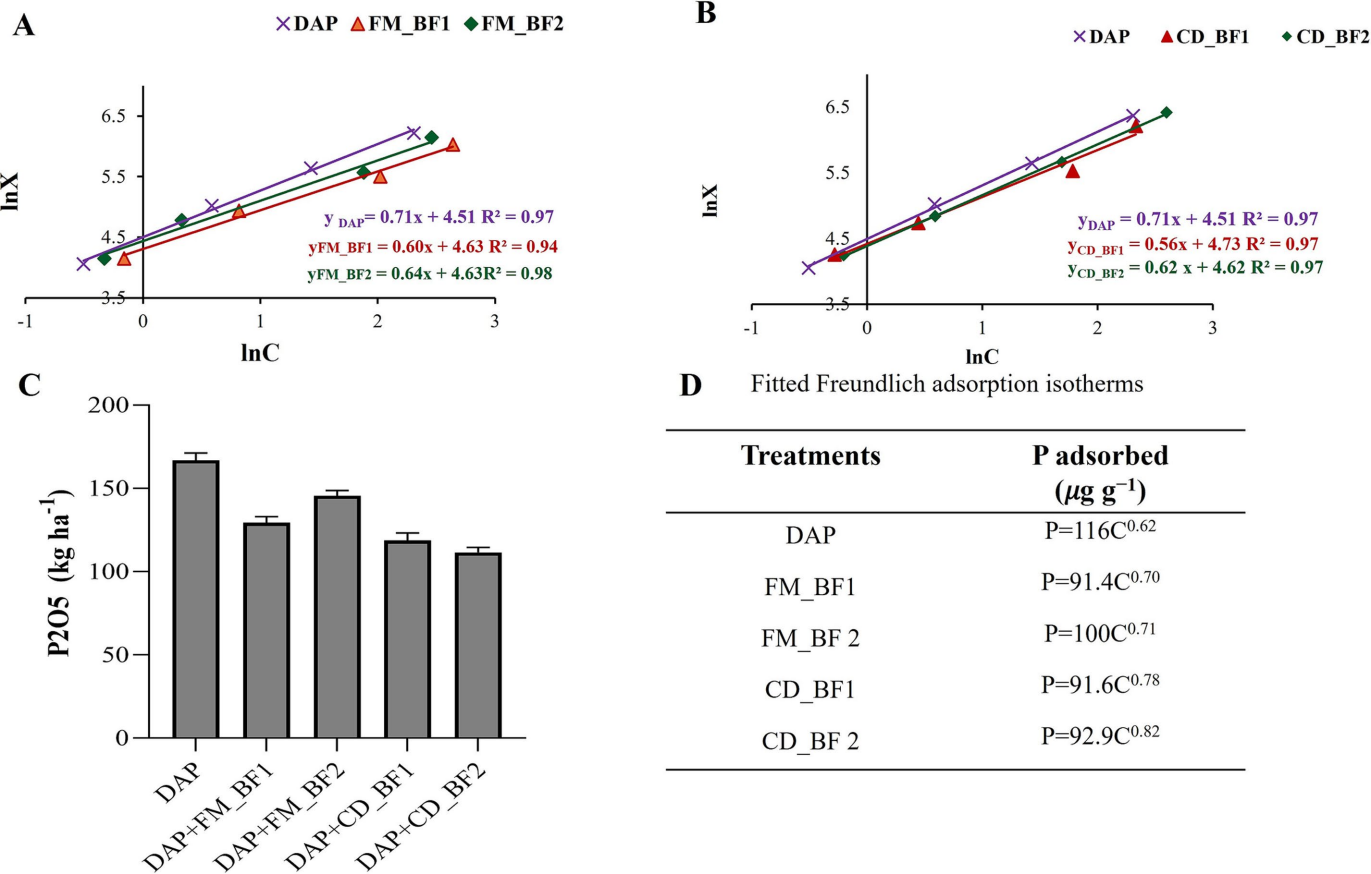
Freundlich isotherm analysis of phosphorus adsorption and fertilizer requirement. **(A,B)** Show Freundlich isotherms for P adsorption of DAP, filter mud-based bioformulations and cow dung-based bioformulations, respectively. Here, X represents the amount of phosphorus adsorbed per unit soil ( $\mathrm{\mu g}\;g^{-1}$ ), and C is the equilibrium phosphorus concentration in solution ( $\mathrm{\mu g}\;mL^{-1}$ ). **(C)** Relationship between soil treatments and P_2_O_5_ requirement (kg ha^−1^) to achieve soil P solution of 0.2 mg L^−1^ and **(D)** fitted Freundlich adsorption isotherms. Treatments: Control as chemical fertilizer DAP = diammonium phosphate, DAP+FM_BF1 (filter mud-based bioformulation at 25 kg/acre applied in combination with DAP), DAP+FM-BF2 (filter mud-based bioformulation at 50 kg/acre applied in combination with DAP 50 kg/acre), DAP+CD-BF1 (cow dung-based bioformulation at 25 kg/acre applied along with DAP), and DAP+CD-BF2 (cow dung-based bioformulation at 50 kg/acre applied along with DAP).

Phosphorus adsorption behavior under different fertilization treatments was evaluated using the Freundlich adsorption model. The adsorption isotherms showed a strong linear relationship between the logarithm of adsorbed phosphorus and equilibrium solution P concentration for both organo-bioformulations, as evidenced by high coefficients of determination (R^2^ ranging from 0.94 to 0.98), indicating an excellent fit of the experimental data to the Freundlich model. The model parameters were statistically significant at *p* ≤ 0.05, further confirming the reliability of the adsorption behavior observed under different treatments (Figures 4A,B).

Phosphorus Requirement as a Function of P Source. It was observed that P adsorption from both organo-bioformulations was lower compared to DAP in soil. Consequently, a lesser quantity of organo-bioformulations was required to achieve the desired P solution, 0.2 mg L^−1^, compared to DAP (Figures 4A,B). The quantity of P₂O₅ (kg ha^−1^) required to achieve a target soil solution phosphorus concentration was calculated from adsorption isotherms for each experimental treatment.

The DAP treatment (control) required the highest amount of P₂O₅ fertilizer (166.96 kg ha^−1^). All treatments amended with organic materials showed a reduced P₂O₅ requirement compared to the control. Among these, the treatments amended with cow dung-based organo-bioformulation (DAP+CD_BF1 and DAP+CD_BF2) demonstrated the lowest requirements, at 118.70 and 111.56 kg ha^−1^, respectively, causing less P adsorption into soil. The treatments amended with filter mud-based organo-bioformulation (DAP+FM_BF1 and DAP+FM_BF2) showed intermediate values of 129.36 and 145.65 kg ha^−1^ (Figure 4C). The use of Freundlich P sorption isotherm, which correlates the concentration of P in soil solution to the amount of P adsorbed in soil, is a better approach in predicting the P fertilizer requirement of a particular soil rather than using soil test. This could be because that soil test provides only information regarding the available P (Bansal et al., 2025) and does not estimate the amount of P fertilizer requirement without calibration for a particular test.

### 3.5 *In planta* evaluation of organo-bioformulation for nutrient uptake and wheat growth promotion

#### 3.5.1 Vegetative growth and yield parameters

**Figure 5:**
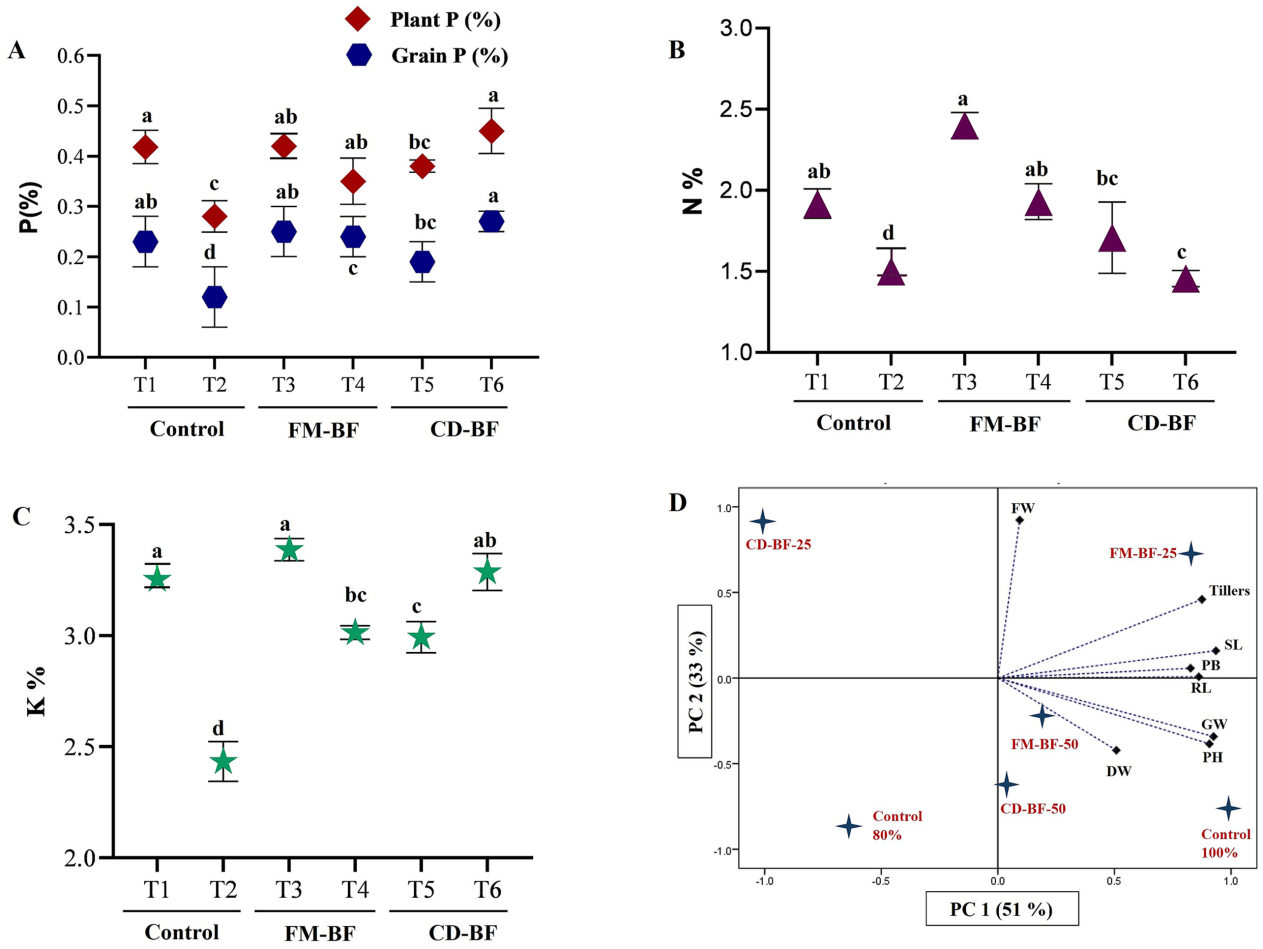
Effect of bioformulations on wheat nutrient content and growth attributes. T1: Control 100%: 100% recommended dose of NPK, T2: Control 80%: 20% reduction in DAP (diammonium phosphate) than the recommended dose, T3:filter mud-based bioformulation (FM-BF) at the rate of 25 kg/acre, T4: filter mud-based bioformulation (FM-BF) at the rate of 50 kg/acre, T5: cow dung-based bioformulation (CD-BF) at the rate of 25 kg/acre, and T6: cow dung-based bioformulation (CD-BF) at the rate of 50 kg/acre **(A)** wheat plant P and grain P content **(B)** plant nitrogen (N) content, **(C)** plant potassium (K) content, and **(D)** PCA of vegetative growth attributes. RL: root length at 65DAG, SL: shoot length at 65 DAS, FW: fresh weight at 65 DAS, PH: plant height at harvest stage, PB: plant biomass at harvest stage, and tillers at harvest stage. Values represented the mean of three biological replicates, with error bars representing standard deviation (St. Dev.). According to least significant difference (LSD), different letters represents statistically significant differences among treatments at *p* < 0.05. Control 100%: 100% recommended dose of NPK without inoculation, Control 80%: 20% reduction in DAP than the recommended dose without inoculation, FM-BF-25: filter mud-based bioformulation at 25 kg/acre, FM-BF-50: filter mud-based bioformulation at 50 kg/acre, CD-BF-25: cow dung-based bioformulation at 25 kg/acre, and CD-BF-50 cow dung-based bioformulation at 50 kg/acre. The letter “a” indicates the highest mean value, with “b” and “c” representing lower means, and combined letters (e.g., ab and bc) showing intermediate groups show no statistically significant difference at *p* = 0.05.

Under net house conditions, the application of both bioformulations significantly enhanced wheat growth parameters relative to the 80% recommended NPK control. Bacteria inoculation had a significant effect on the length of roots, shoot length, fresh and dry weight of the plant at 65 DAS, and the harvest stage. Treatment T3 (FM-BF at 25 kg/acre) showed the most pronounced effect, with an increase in plant height (26 and 11%) at 65 DAS, plant biomass (24%), and grain yield (16%) at the harvest stage. T4 (CD-BF at 50 kg/acre) resulted in 8% increase in height, 13% in plant biomass, and 14% in grain yield at the harvest stage (Table 3). LSD analysis confirmed these effects to be statistically significant (*p* < 0.05). Bioformulation application significantly improved nutrients [nitrogen (N), phosphorus (P), and potassium (K)] uptake in wheat (Figures 5A–C). At 65 DAS, straw P was found higher in T6 (CD-BF = 50 kg/acre), followed by T3 (FM-BF = 25 kg/acre), with an increase of 0.47 and 0.42%, respectively, compared to the control (80%). Similarly, at the harvest stage, grain P was significantly higher in T6 (0.3%), followed by T3 (0.28%) as compared to the 80% control (0.12%). Chlorophyll content of wheat plants was evaluated at 65 DAS by using a SPAD meter (Table 3). Principle component analysis (PCA) indicates a positive correlation between filter mud-based organo-bioformulation and root length, shoot length, fresh weight, grain weight at 65 DAS, tillers, and plant height at harvest stage, PB: plant biomass at harvest stage, and tillers’ grain weight, height, and soil available P as compared with both 100 and 80% controls. The two principal components generated up to 84% of the variance on the x-axis (PC 1 = 51%) and y-axis (PC 2 = 33%) (Figure 5D).

**Table 3:** Effect of FM-BF and CD-BF on plant growth parameters at 65 DAS and harvest stage in pot experiment under net house conditions.

		At 65 days after sowing^1^					At harvest stage^2^			
Treatments	Plant height (cm)	Root length (cm)	Fresh weight (g)	Dry weight (g)	Chlorophyll content	Viable count cfu/g	Plant height (cm)	Plant weight (g pot^−1^)	Tillers per plant	100 grains weight (g)
Control 100%	54.6 ± 2.5 bc	11.2 ± 2.4 b	17.0 ± 0.7 bc	5.6 ± 0.6 b	85.8 ± 0.5a	1.8×10^7^	86.3 ± 3.8 ab	15.3 ± 2.0 ab	4 ± 0.03 a	4.6333 ± 0.40 abc
Control 80%	48.6 ± 3.7 d	8.0 ± 1.15 d	13.4 ± 2.6 d	4.0 ± 0.4 d	80.2 ± 1.4 c	1.7×10^7^	79 ± 1.0 d	13 ± 1.03 c	3.3 ± 0.5 b	3.9333 ± 0.20 d
FM-BF 1	65.3 ± 2.5 a	13.2 ± 1.0 a	22.6 ± 3.1 a	6.3 ± 1.3 a	86.9 ± 0.6 a	2.3×10^7^	88.6 ± 2.2 a	16.2 ± 1.2 a	4.1 ± 0.6 a	4.8667 ± 0.15 a
FM-BF 2	57.6 ± 2.6 b	11 ± 0.5 b	18.9 ± 1.7 bc	5.8 ± 0.5 b	83.6 ± 1.0 ab	2.5×10^7^	85.3 ± 4.5 b	14.2 ± 1.6 b	3.6 ± 0.17 ab	4.4333 ± 0.15 b
CD-BF 1	50.6 ± 2.1 c	9.2 ± 1.03 c	13.0 ± 2.6 cd	4.4 ± 0.7 cd	82.9 ± 1.5 ab	2.2×10^7^	82 ± 3.1 c	13.6 ± 0.6 bc	3.6 ± 0.06 ab	4.2333 ± 0.41 bc
CD-BF 2	59.0 ± 2.0 b	10.1 ± 1.2 bc	20.2 ± 2.8 ab	6.2 ± 0.4 ab	84.4 ± 1.6 ab	2.8×10^7^	86.3 ± 4.4 ab	15 ± 1.0 ab	3.3 ± 1.2 b	4.7333 ± 0.50 ab

Effect of filter mud-based bioformulation (FM-BF) and cow dung-based bioformulation (CD-BF) on different wheat growth parameters at (1) 65 DAS and (2) harvesting stage in a pot experiment, Faisalabad. The values displayed are the mean value of three replicates. ± represents standard deviation (SD). Significant differences among treatments (*p* < 0.05) are indicated by different letters. Control 100%: 100% recommended dose of NPK without inoculation, Control 80%: 20% reduction in DAP than the recommended dose without inoculation, FM-BF-1: filter mud-based bioformulation at 25 kg/acre, FM-BF-2 filter mud-based bioformulation 50 kg/acre, CD-BF: cow dung-based bioformulation at 25 kg/acre, and CD-BF-2 cow dung-based bioformulation at 50 kg/acre.

#### 3.5.2 Nutritional content

Bioformulation application significantly improved nutrients (nitrogen (N), phosphorus (P), and potassium (K)) uptake in wheat (Figure 5). At 65 DAS, plant P was found higher in T6 (CD-BF = 50 kg/acre), followed by T3 (FM-BF = 25 kg/acre), with an increase of 35 and 32%, respectively, compared to the control (80%). At the harvest stage, grain P was significantly higher in T6 (0.3%), followed by T3 (0.28%), as compared to the 80% control (0.12%) (Figure 5A). Wheat straw also significantly enhanced N (2.4%) (Figure 5B) and K (3.3%) content in inoculated plants as compared to the 80% control uninoculated plants (Figure 5C).

**Figure 6:**
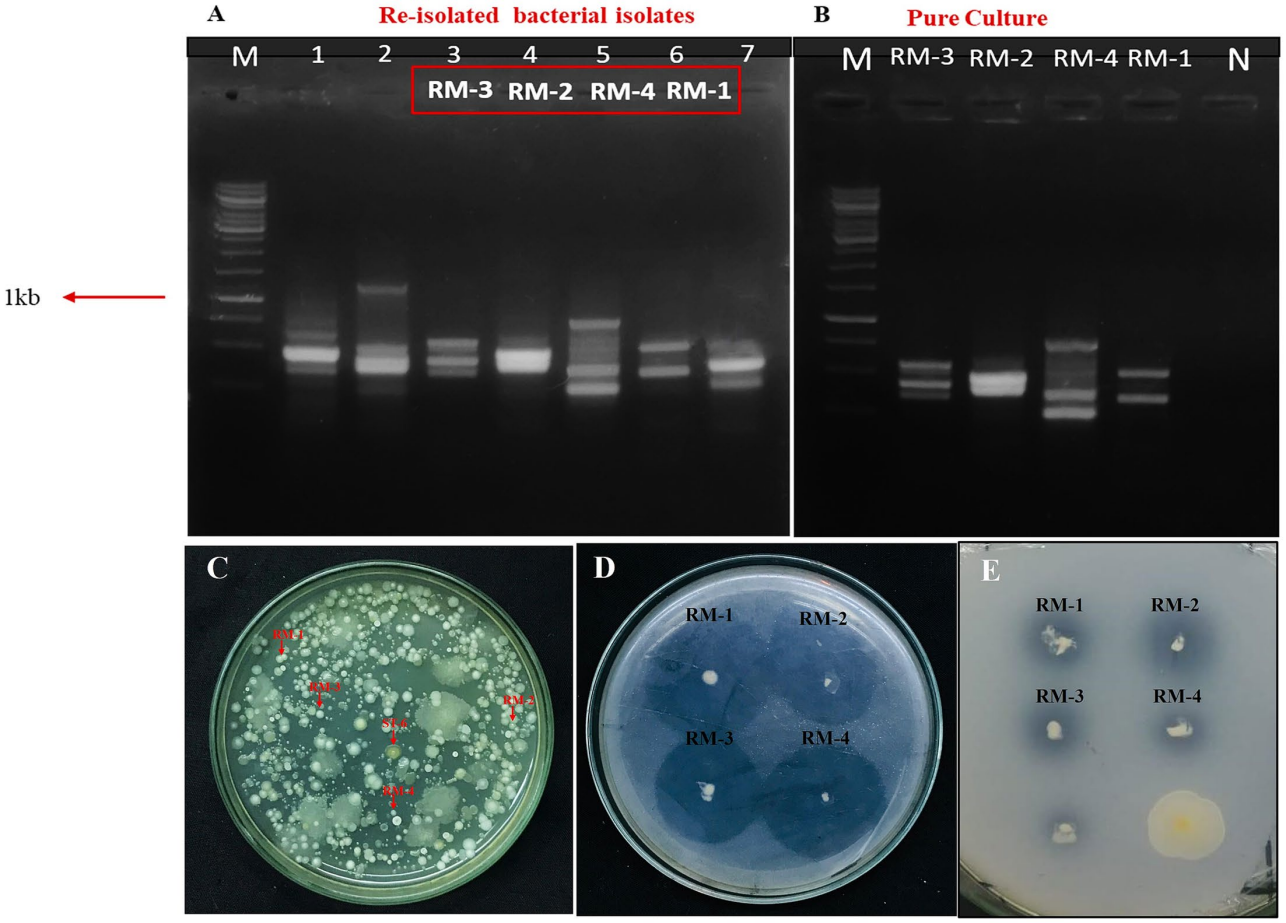
Re-isolation of inoculated PSB colonies. Re-isolation of inoculated PSB colonies. **(A)** BOX-PCR patterns of pure cultures of PSB isolated from wheat rhizosphere. M = 1 kb DNA ladder; RM-3, RM-2, RM-4, and RM-1 are indicated in the respective lanes. **(B)** BOX-PCR patterns of re-isolated colonies from soil. M = 1 kb DNA ladder; lanes show re-isolated RM-3, RM-4, RM-2, and RM-1. **(C)** Plate showing re-isolated colonies of RM-1, RM-2, RM-3, and RM-4. **(D)** Zinc solubilization was confirmed by the development of a zone around PSB colonies grown on an agar medium containing ZnO**. (E)** NBRIP agar plate demonstrating phosphate solubilization zones formed by re-isolated colonies of RM-1, RM-2, RM-3, and RM-4.

The survival of inoculated PSB was assessed using the viable count method. Morphological traits of inoculated PSB enabled the detection of the inoculated rhizospheric bacteria, indicating their rhizosphere competence. BOX-PCR further validated the identification of inoculated phosphate-solubilizing bacteria. Furthermore, BOX-PCR analysis confirmed that re-isolated colonies were identical to pure cultures (Figure 6).

#### 3.5.3 Microplot and field experiment to evaluate agronomic and yield parameters under natural conditions

**Figure 7:**
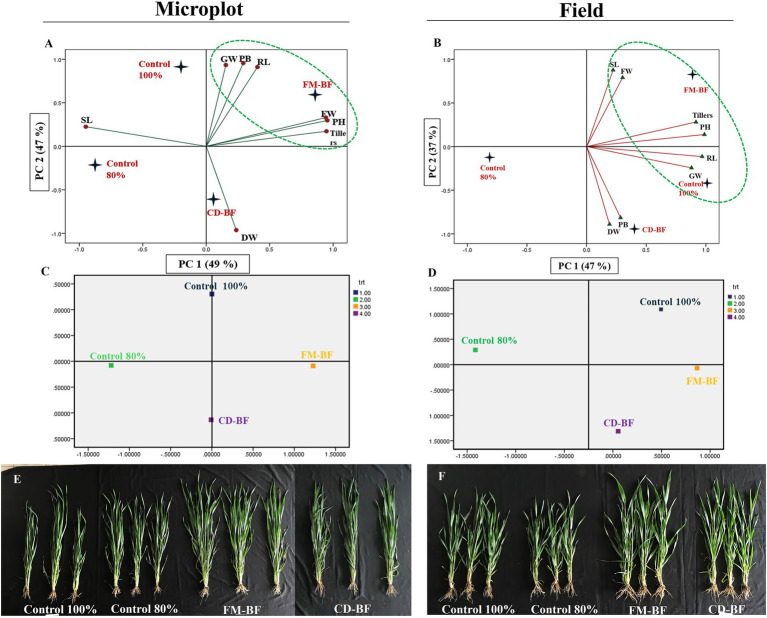
Principal component analysis (PCA) and visual comparison of wheat growth performance under different bioformulation treatments. **(A,C,E)** Show microplot experiment, and **(B,D,F)** show field condition data. **(A,B)** PCA biplots showing the association among vegetative growth traits and treatments under two experimental conditions. PC1 and PC2 explained 49 and 47% of the total variance in **(A)** and 47 and 37% in **(B)**, respectively. Growth attributes included RL (root length at 65 DAS), SL (shoot length at 65 DAS), FW (fresh weight at 65 DAS), DW (dry weight), PH (plant height at harvest), PB (plant biomass at harvest), GW (grain weight), and number of tillers at harvest. Treatments comprised Control 100% (recommended NPK), Control 80% (20% reduced DAP), FM-BF (filter mud-based bioformulation at 25 kg/acre), and CD-BF (cow dung-based bioformulation at 25 kg/acre). **(C,D)** PCA score plots illustrating treatment clustering and their relative contribution to growth variability. **(E,F)** Representative images of wheat plants under respective treatments, demonstrating morphological differences in root and shoot development at the harvest stage.

The microplot experiment was conducted under net house conditions and reinforced the pot experiment findings. Application of FM-based organo-bioformulation and CD-based organ bioformulation significantly improved wheat growth parameters compared to the 80% control. T3 (FM-BF = 25 kg/acre) significantly improved plant height (9%), root length (16%) at 65 DAS, plant biomass (20%), and 1,000-grain weight (16%) at harvest stage over the 80% control. T4(CD-BF = 25 kg/acre) also improved growth, plant height by 16 and 4%, root length by 13%, and grain yield by 12% at 65 DAS and harvest stage (Table 4). Principal component analysis (PCA) indicates a positive correlation between filter mud-based organo-bioformulation and root length, fresh weight, at 65 DAG, tillers, grain weight, and plant height at harvest stage, as compared with both 100 and 80% controls. The two principal components contributed up to 96% toward variance on the x-axis (PC 1 = 47%) and y-axis (PC 2 = 49%) (Figure 7A).

**Table 4:** Effect of FM-BF and CD-BF on plant growth parameters at 65 DAS and harvest stage in microplot experiment and field conditions.

		At 65 days after sowing^1^						At harvest stage (per plot)^2^				
	Treatments	Plant height (cm)	Root length (cm)	Fresh weight (g)	Dry weight (g)	Chlorophyll content	Viable count (cfu/g)	Plant height (cm)	Total weight (kg)	Grain weight (g)	1,000 grain weight (g)	Tillers
Microplot	Control l00%	63.6 ± 1.5 b	12.6 ± 1.5 ab	50.3 ± 1.5 b	6.8 ± 0.6 b	83.4 ± 0.5 b	1.8×10^6^	101 ± 4.3 ab	2.057 ± 36 ab	821.67 ± 27 ab	42.33 ± 1.4 a	407.33 ± 47 ab
	Control 80%	58.6 ± 2.0 c	8.7 ± 0.25 c	43.2 ± 0.7 c	5.4 ± 0.7 c	80.4 ± 0.63 c	1.3×10^6^	93 ± 9.07 c	1.594 ± 45 c	783.33 ± 25 c	35.00 ± 2.8 b	343.00 ± 37 b
	FM-BF	72.6 ± 2.5 a	10.4 ± 0.3 bc	54.2 ± 0.8 a	8.2 ± 0.92 a	85.6 ± 0.9 a	2.4×10^7^	103 ± 5.50 a	2.283 ± 17 a	888.00 ± 50 a	42.33 ± 1.42 a	432.0 ± 36 a
	CD-BF	65.0 ± 0.5 b	14 ± 0.85 a	45 ± 1.03 c	6.3 ± 0.91 bc	83.5 ± 0.6 b	2.7×10^8^	97 ± 2.08 bc	1.845 ± 53 b	802.67 ± 15 b	40.00 ± 2.6 ab	383.67 ± 35 ab
Field	Control l00%	60.0 ± 2.6 b	11.8 ± 0.9 b	38.3 ± 0.8 ab	8.2 ± 0.6 b	82.4 ± 0.8 b	2.5×10^7^	97 ± 4.3 ab	27.37 ± 19 ab	2,620 ± 94 ab	37.00 ± 2.0 ab	23.1 ± 24 a
	Control 80%	54.6 ± 3.2c	7.3 ± 0.5d	30.7 ± 0.7 c	4.6 ± 0.3 c	78.8 ± 1.4 c	1.5×10^6^	90 ± 9.0 c	19.04 ± 21 c	2,220 ± 90 c	25.33 ± 3.51 c	18.3 ± 23 b
	FM-BF	67.3 ± 2.08 a	14.4 ± 0.8a	41.4 ± 0.8 a	9.7 ± 1.2 a	85.5 ± 1.1 a	2.6×10^7^	100 ± 5.5 a	29.32 ± 18 a	2,750 ± 87 a	40.66 ± 2.6 a	22.08 ± 40.0 a
	CD-BF	62.0 ± 2.0 b	9.2 ± 1.1 bc	35.2 ± 1.3 b	7.3 ± 0.7 bc	82.3 ± 0.8 b	3.8×10^7^	95 ± 2.0 b	25.83 ± 11 b	2,370 ± 125 b	34.66 ± 3.0 b	21.43 ± 17.2 ab

Effect of FM-BF and CD-BF on various wheat growth parameters at 65 DAS (1) and harvesting stage (2) in a microplot experiment, Faisalabad. Values indicated the average of three replicates. Standard deviation (SD) is represented by the error bar. Significant differences among treatments (*p* < 0.05) are indicated by different letters. Control 100%: 100% recommended dose of NPK without inoculation, Control 80%: with no inoculation, the recommended dose of DAP is reduced by 20%, FM-BF at 25 kg/acre, and CD-BF at 25 kg/acre.

Under natural field conditions, the application of both organo-bioformulations sustained their performance and significantly improved plant growth parameters at 65 DAS and the harvest stage. T3 (FM-BF = 2 kg/acre) increased plant height by 10%, total biomass by 14%, and grain yield by 17% at the harvest stage. CD-BF (T4 at a rate of 25 kg/acre) also improved these traits by 5, 4, and 6%, respectively, as compared to the 80% control (Table 4). The LSD test confirmed significance (*p* < 0.05), highlighting the robustness of both treatments across scales. Principal component analysis (PCA) indicates a positive correlation between filter mud-based organo-bioformulation and root length, shoot length, fresh weight, at 65 DAS, tillers, grain weight, and plant height at harvest stage, as compared to uninoculated 100 and 80% controls. The two principal components generated up to 84% toward variance on the x-axis (PC 1 = 47%) and y-axis (PC 2 = 37%) (Figure 7B).

#### 3.5.4 Physiological parameters

The effect of both organo-bioformulations on the chlorophyll content of wheat plants was recorded using an SPAD meter. 6.4% increase in FM-BF-treated plants and 3.8% in CD-BF over the 80% control were recorded. Under filed conditions, T4 (FM-BF = 25 kg/acre) showed an 8.5% increase and 4.4% in CD-BF (25 kg/acre) in treated plots compared to the 80% NPK control (Table 4).

#### 3.5.5 Post-harvest nutritional analyses

**Figure 8:**
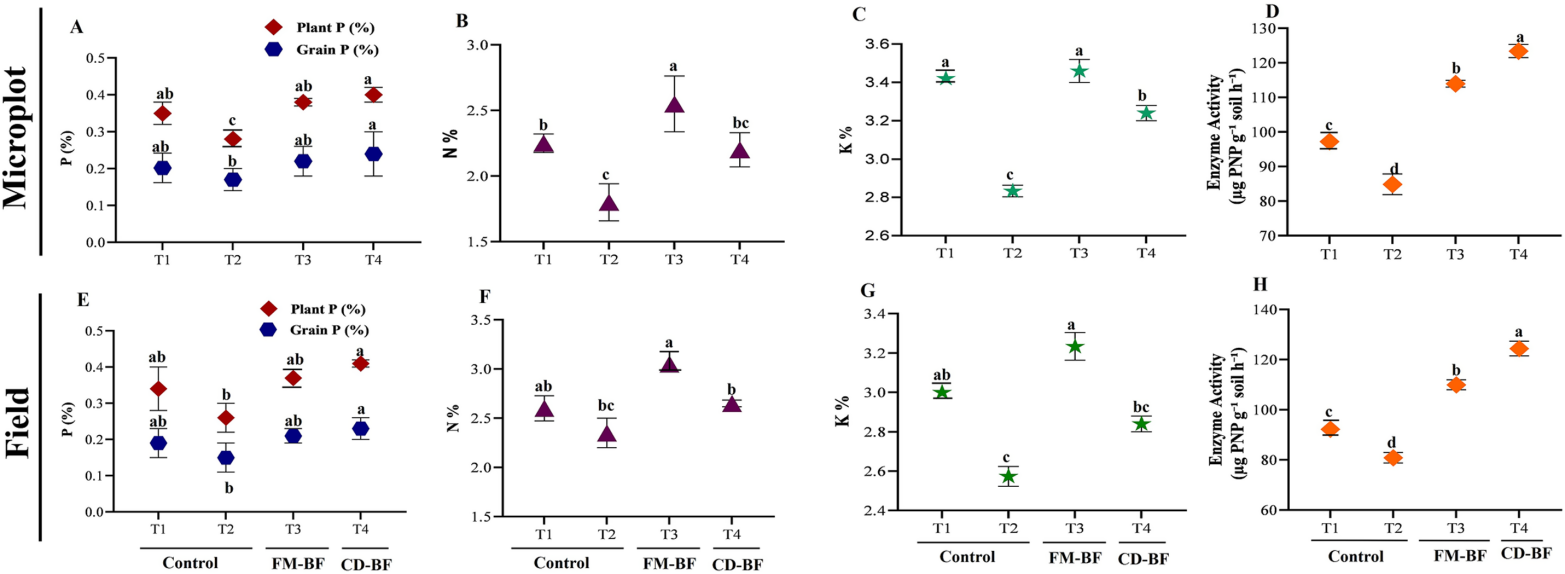
Effect of bioformulation treatments on wheat nutrient content and soil phosphatase activity under microplot and field conditions. Treatments included T1: Control 100%: 100% recommended dose of NPK without inoculation, T2: Control 80%: 20% reduction of DAP than the recommended dose without inoculation, T3: FM-BF: filter mud-based bioformulation at 25 kg/acre, T4: CD-BF: cow dung-based bioformulation 25 kg/acre. Under microplot experiment, **(A)** wheat plant P and grain P content, **(B)** plant nitrogen (N) content, and **(C)** plant potassium (K) content. **(D)** Soil phosphatase activity is indicated by the release of p-nitrophenyl/h. Under field conditions, **(E)** shows wheat plant P and grain P content, **(F)** plant nitrogen (N) content, and **(G)** plant potassium (K) content. **(H)** Soil phosphatase activity is indicated by the release of p-nitrophenyl/h. Error bars on each treatment represent the standard deviation (SD). Significant differences among treatments (*p* < 0.05) are indicated by different letters.

In the microplot experiment, both organo-bioformulations significantly improved plant nutrient (NPK) content and grain P. Application of CD-BF significantly enhanced plant P (38%), N(22%), and K(18%) content in T4 (CD-BF = 25 kg/acre), followed by FM-BF (T3) improved straw P (0.4%), N (40%), and K (34%) content as compared to the 80% control. At the harvest stage, grain P reveals significant improvement in T4 (CD-BF = 25 kg/acre) as compared to the 80% control (Figures 8A,B).

Under field conditions, both organo-bioformulations had a pronounced effect on wheat plant nutrient (NPK) content and grain P at 65 DAS and during the harvest stage. Phosphorus content of wheat plant at 65 DAS was significantly improved in T4 (CD-BF = 25 kg/acre), with the recording value of 0.43%, followed by T3 (FM-BF 25 kg/acre) with a P content of 0.41% (Figure 8C). Nitrogen content increased by 30% and potassium by 26%, compared to the 80% control (Figure 8D), while at the harvest stage, grain P was higher in CD-BF (T4 = 25 kg/acre) by 21%, relative to the 80% control (Figure 8C).

#### 3.5.6 Estimation of soil phosphatase activity

Soil phosphatase activity was determined using p-nitrophenyl as substrate. Light pink color was developed when p-nitrophenyl reacted with phosphatase. The highest phosphate activity (123.4 μmoles/g of soil/h) was observed in treatment 4 (CD-BF = 25 kg/acre), and treatment 3 (FM-BF = 25 kg/acre) showed phosphatase activity (113.22 μmoles/g of soil/h). Organo-bioformulations showed significant results (*p* < 0.05) compared to the control at 80% (Figure 8D).

## 4 Discussion

Modern agricultural systems continue to rely extensively on inorganic/synthetic fertilizers (Albano et al., 2023; Prout et al., 2021), leading to loss of organic matter, hindering water infiltration, soil microbial activity, soil fertility, altering the soil nutrient cycle, i.e., mineralized (P), and causing environmental hazards (Adnan et al., 2022). Consequently, the development of eco-friendly, low-cost, and efficient organic bioformulations offers an alternative and sustainable strategy for modern agriculture (Aslam et al., 2024). Thus, this study was designed to investigate organo-bioformulations incorporating phosphate-solubilizing PGPR with organic carriers and quantified their P release pattern alongside DAP using the Freundlich equation and their application in Pakistan staple crop wheat.

Five efficient phosphate-solubilizing bacteria were isolated from drought-stressed regions of South Punjab, Pakistan (Table 1) and characterized them for plant growth-promoting traits (Table 1). These PSB can solubilize inorganic phosphate source TCP (tricalcium phosphate) up to 234 μL/mL to 448 μL/mL, with a decrease in pH up to 4.1 (Figure 1). The reduction in pH is likely due to the production of organic acids which favor phosphate solubilizing (Sharma et al., 2013; Kim et al., 2019; Ahemad and Kibret, 2013).

Thus, the combination of different plant growth-promoting traits in selected PSB makes them potentially beneficial as biofertilizers and reduces reliance on chemical fertilizers for crop production. Roshani et al. have observed that application of bacterial consortia spraying increased seed germination and vigor index of wheat seedlings (Roshni et al., 2020). Kumar et al. documented an increase in wheat growth upon the application of an indigenous rhizobacteria consortium (Kumar et al., 2020). These early plant growth improvements can be attributed to multiple factors, particularly the production of IAA and the increased phosphorus availability. The P-solubilizing activity of the bacteria was probably responsible for P availability for germinating seeds and young seedlings while facilitating rapid early growth (Zaidi et al., 2009), and bacterial IAA production improved root and shoot elongation in young seedlings (Glick, 2012).

Gram staining and light microscopy revealed that the majority of isolates were rod-shaped and Gram-negative. The 16S rRNA gene sequencing identified RM-1, RM-2, RM-3, RM-4, and RM-5 were Gram-negative (Table 1).

The isolated strains showed considerable zinc solubilization ability. The ability of PSB to solubilize both phosphate and zinc implies their potential to simultaneously address several nutrient deficiencies in alkaline soil (Goteti et al., 2013; Nadeem et al., 2024). Phosphorus and zinc solubilization is related to the production of growth hormone indole acetic acid that can lead to a reduction in pH of the rhizosphere directly by the action of root exudate release, such as malic acid, oxalic acid, and citric acid, thus increasing the availability of P and Zn (Etesami and Adl, 2020). Furthermore, IAA is recognized for promoting root elongation and growth, which improves nutrients and water uptake in plants (Spaepen and Vanderleyden, 2011). All bacterial isolates demonstrated nitrogen fixation potential, contributing to plant nitrogen content essential for chlorophyll and protein synthesis (Goswami et al., 2016). Many phosphate-solubilizing bacteria (PSB), such as *Pseudomonas* and *Paenibacillus*, also possess nitrogen-fixing abilities through nif gene-mediated nitrogenase activity, converting atmospheric nitrogen into plant-available forms, thereby improving wheat growth, root length, nitrogen uptake, and yield under nutrient-deficient soils (Upadhayay et al., 2022; Bhattacharyya and Jha, 2012). ACC deaminase-producing bacteria reduce plant ethylene levels under stress conditions. This reduction prevents stress-induced growth inhibition, improving root elongation, biomass, chlorophyll content, and yields in wheat, especially under salinity and drought conditions (Nadeem et al., 2010). In addition to having multiple plant growth-promoting traits, these isolates were capable of producing siderophores, which enhance iron availability by chelating Fe^3+^ and improve chlorophyll content, grain iron concentration, and overall crop yield (Nadeem et al., 2010; Yue et al., 2022).

For the development of two organo-bioformulations, a consortium of elite bacterial isolates was used with different organic carrier materials such as filter mud and cow dung, each supplemented with 5% sodium alginate as an adhesive. While previous studies have largely emphasized the efficacy of microbial inoculants, limited attention has been given to the selection and optimization of carrier-based bioformulations (Koskey et al., 2021; Soumare et al., 2020). The efficacy and shelf life of bioinoculants are strongly influenced by the physicochemical properties of carrier materials and the adhesives employed, which play a critical role in ensuring inoculum survival prior to field application (Soumare et al., 2020). Carrier materials provide protective niches, buffer against environmental stress, and enhance microbial persistence by reducing predation pressure (Yan et al., 2020). It is consistent with the previous study as sodium alginate was selected due to its biocompatibility, ability to enhance microbial survival, facilitate controlled release, and promote rhizosphere colonization (Van Elsas et al., 1992; Tamreihao et al., 2016). Alginate-based encapsulation has been reported to improve seed germination by 70–80% and increase root and shoot lengths by 60–70%, along with significant enhancements in biomass production compared to untreated controls (Yan et al., 2020).

Physicochemical characterization of both organo-bioformulations (FM-BF and CD-BF) revealed distinct differences between nutrient composition and organic matter quality (Table 2), a factor that likely influenced their soil functional behavior and P-release pattern in soil and plant. Thus, carrier materials with balanced or low heavy metal content are viable candidates for the development of organo-bioformulation (Yan et al., 2020).

Shelf-life analysis revealed that both formulations maintained survival of inoculated PSB populations up to 90 days post-inoculation; however, FM-BF consistently supported higher survival of inoculated PSB load up to 90 DPI (Figure 3). This aligns with a prior study that filter-mud formulation maintained substantial bacterial survival over time, likely due to its nutrient-rich organic matrix and moisture-retentive properties, which are known to buffer microbial cells against desiccation and nutrient stress during storage (Aslam et al., 2024). Previous study has demonstrated that sugarcane mill mud (filter mud) is enriched with organic carbon, nitrogen, and nutrient minerals, making it a suitable substrate for supporting microbial activity and soil biological functions when used as a soil amendment or carrier material (Uchimiya et al., 2022). The enhanced shelf life of FM-BF indicates that filter mud provides a more favorable microenvironment for PSB survival and is a potential candidate for stable bioinoculant delivery without the need for specialized storage conditions (Soumare et al., 2020).

Moreover, a soil microcosm study was performed that evaluated the efficacy of organo-bioformulation in mobilizing soil fixed phosphorus and enhancing soil available P up to 35% as compared to traditional chemical phosphorus fertilizers. These findings collaborate with the previous finding of Zaidi et al. (2017), which demonstrated that PSB can progressively enrich extractable P from different P sources even in calcareous environments, highlighting their potential to counteract P fixation and improve P nutrition when combined with organic amendments (Adnan et al., 2017).

Phosphorus adsorption behavior was well described by the modified Freundlich equation, as indicated by high coefficients of determination (*R*^2^ ≥ 0.84), confirming strong conformity between experimental data and the model. Similar observations have been reported by (Sarfraz et al., 2009), who demonstrated that Freundlich adsorption parameters are primarily influenced by solution P concentration rather than incubation time or temperature. The Freundlich adsorption model effectively described the relationship between equilibrium P concentration and P adsorption in the studied soils, indicating its suitability for evaluating P sorption behavior under different bioformulation treatments. Therefore, the use of Freundlich isotherms represents a time-efficient and precise method for optimizing P fertilizer inputs.

The reduced P adsorption observed under organo-bioformulations can be attributed to the presence of organic matter and phosphate-solubilizing bacteria (PSB). Previous studies have shown that organic amendments activate soil P and reduce adsorption by masking P sorption sites through organic ligands and decomposition products such as carbohydrates (Zhao et al., 2014; Zhang et al., 2005). These organic compounds also increase P saturation at residual adsorption sites, thereby lowering phosphate binding energy (Xiaoqi and Rukun, 1991). Furthermore, PSB secretes low-molecular-weight organic acids that compete with phosphate ions for adsorption sites and dissolve Ca-bound phosphates, leading to decreased P fixation (Shi et al., 2017). Li et al. (2014) reported that PSB application reduced maximum P adsorption capacity and adsorption constants while enhancing P desorption capacity, which aligns well with the outcomes of the present study.

After formulating and evaluating the shelf life of both organo-bioformulations, FM-BF (filter mud-based) and CD-BF (cow dung-based), their agronomic potential was further investigated through an experiment conducted under a net house (pot and microplot) and field conditions. Under the net house (pot and microplot) experiment, FM-BF had a significant impact on wheat growth parameters, including plant height, root length, chlorophyll content, and grain yield. These improvements were associated with increased plant nitrogen (N) and potassium (K) content. In contrast, CD-BF increased substantially plant P uptake, grain P, and soil phosphatase activity (123.4 μmol/g soil/h), indicating its efficacy in P solubilization and cycling. The findings are consistent with prior research demonstrating increased rice growth (Tahir et al., 2013) and wheat grain yield (Suleman et al., 2018) under agro-ecological conditions when seed is pelleted with filter mud and PSB. As a result, filter mud (FM) can serve as an effective carrier for the PSB, enhancing soil nutrient availability and wheat grain output and offering a sustainable, eco-friendly strategy for future agricultural production. Multiple PSB features have a direct impact on plant bioavailability and P uptake in plants. By secreting bacterial organic acids, PSB improved the plants’ nutrient-sensing capacity (Yahya et al., 2021). PSB regulates root architectural morphology, alleviating stress by mobilizing soil phosphorus and facilitating plant P uptake. This tripartite approach may result in better crop vigor and crop yield. Furthermore, field trials validated these findings that organo-bioformulation elevated wheat grain yield (17%), plant biomass (14%), plant height (10%), and chlorophyll content (8%) over 80% control. These results align with several prior studies that reported enhanced wheat growth under FM and PSB-based treatments (Suleman et al., 2018; Tahir et al., 2013; Yahya et al., 2022). Moreover, both organo-bioformulation contributed to enhancing wheat essential nutrients. NKP supported by Malaysian research reported that integration of press mud biofertilizer improved N, P, and K uptake by 85–384 mg, 3–12 mg, and 25–82 mg, respectively, emphasizing its role in efficient nutrient delivery (Yue et al., 2022).

Viable count and re-isolation studies further validated the successful competence and persistence of the inoculated PSB isolates in the wheat rhizosphere. Recovered strains retained plant growth-promoting characteristics, affirming their survival and functional activity within the wheat rhizosphere (Hakim et al., 2021; Lopes et al., 2021).

Our findings demonstrate the potential of carrier-based organo-bioformulations to improve crop productivity by enhancing nutrient acquisition and mobilization of soil-bound phosphorus, thereby enhancing the efficiency of applied chemical phosphorus. The application of locally sourced organic carriers such as filter mud and cow dung not only boosts plant nutrient absorption and plant biomass but also stimulates soil biological activity. This study underscores the role of bioformulations in soil nutrient management strategies aimed at reducing dependence on traditional chemical fertilizers. By providing a cost-effective, eco-friendly alternative, this research offers promising prospects for long-term soil fertility restoration and resilient wheat production.

## 5 Conclusion

The present study integrates the development of organo-bioformulation with mechanistic evaluation of phosphorus sorption dynamics to better understand how microbial bioformulations influence P availability and fertilizer requirements in calcareous soils. The formulation and application of organo-bioformulations comprised of organic carrier materials, alginate as adhesive, and well-characterized PSB significantly improved wheat growth parameters, P nutrition, and grain yield as compared with the control under net house conditions and field conditions. Sorption analysis using the Freundlich model effectively quantified soil P adsorption and demonstrated that the fertilizer requirements can be more accurately determined through sorption modeling rather than conventional soil test *p* values. Moreover, using microbial-based biofertilization instead of synthetic chemical fertilizer could result in less P adsorption and fixation in calcareous soils and reduce the consumption of chemical fertilizer. However, multi-location and long-term field trials need to be further verified for their agronomic performance under diverse agro-climatic conditions before formulating countrywide recommendations.

## Data Availability

The datasets presented in this study can be found in online repositories. The names of the repository/repositories and accession number(s) can be found in the article/supplementary material.

## Footnotes

1
http://www.ncbi.nlm.nih.gov
